# Electric bicycles-related orthopedic injury spectrum: retrospective analysis of 1,735 cases (2020–2025)

**DOI:** 10.1186/s13018-026-06965-3

**Published:** 2026-06-06

**Authors:** Zhi Wang, Keyi Zhao, Junxian Zheng, Congcong Chen, Gan Zhang, Xiaosong Chen

**Affiliations:** Department of Orthopaedics, the 901st Hospital of the Joint Logistic Support Force of the People’s Liberation Army of China, Anhui, 230031 China

**Keywords:** Electric bicycles, Orthopedic injuries, Epidemiology, Trauma management

## Abstract

**Background:**

The rapid adoption of electric bicycles (EB) has led to a significant increase in related injuries, posing a growing public health challenge. In Anhui Province, China, EB-related orthopedic injuries represent a major component of traffic trauma burden. However, systematic data on injury patterns, anatomical distribution, and demographic variations remain limited, hindering optimized clinical management. This study aims to characterize the clinical spectrum of orthopedic injuries associated with EB that necessitate surgical management.

**Methods:**

This single-center retrospective cohort study analyzed data from the Hospital Information System (HIS) for patients with EB-related orthopedic injuries between January, 2020, and December, 2025. Among 3,412 vehicle-related injuries, 1,735 cases met inclusion criteria. Injury types were classified into six categories (e.g., fractures, dislocations), and anatomical sites were categorized into 16 regions. Statistical analyses included descriptive statistics and chi-square tests to identify factors associated with severe injuries.

**Results:**

The study included 1,735 patients (59.20% male; mean age 48.65 ± 15.73 years), with a bimodal age distribution peaking in the 31–44 and 45–59 groups. Fractures predominated (85.01% of cases), followed by combined injuries such as open fractures with soft tissue damage (4.67%). The most frequent anatomical sites were the clavicle, tibiofibula, and hand/foot. Female patients were significantly older than males (95% CI: 3.44, 6.38; *p* < 0.001), and young males had higher injury rates.

**Conclusions:**

EB-related orthopedic injuries predominantly affect middle-aged and elderly populations (1,735 patients; mean age 48.65 ± 15.73 years, bimodal peaks at 34.65 ± 9.41 years and 57.28 ± 6.72 years), with fractures accounting for 85.01% of cases and combined trauma (e.g., open fractures with soft tissue damage) representing 4.67%. The clavicle, tibiofibula, and hand/foot are the most commonly injured sites. These findings provide foundational insights for orthopedic clinical practice pertaining to EB-related injuries, suggesting that age-stratified triage protocols and prioritized evaluation of high-risk anatomical sites (clavicle, lower limbs) warrant further investigation to optimize resource allocation and patient outcomes in clinical settings. However, this study has several limitations, including its single-center retrospective design, absence of severity validation scores, and insufficient data on protective measures/devices/follow-up and so on. Therefore, prospective multicenter studies are warranted to validate and optimize clinical practice.

**Electronic supplementary material:**

The online version of this article (10.1186/s13018-026-06965-3) contains supplementary material, which is available to authorized users.

## Background

The growing popularity of electric bicycles (EB), driven by their convenience for short trips and their ability to bypass traffic congestion, has resulted in a sharp rise in their usage [1–3]. However, this surge has also given rise to increasingly prominent social challenges. In China, in 2020, fatalities among EB riders constitute approximately 13.51% of total traffic-related deaths, while injuries account for around 17.43% of all traffic accident casualties [4]. In these traffic accidents involving EB, the ratio of fatalities attributable to head injuries versus orthopaedic injuries (including rib fractures) is approximately 6:4. Furthermore, Anhui Province, where the author is based, ranks among the highest in the nation with respect to the incidence of EB-related accidents [4].

EB-related injuries are significantly more prevalent than fatalities resulting from such accidents, encompassing harm sustained by riders and passengers as well as injuries involving third parties due to the operation of these vehicles [4–7]. These injuries are increasingly recognized as a major contributor to orthopedic trauma [8]. However, existing research remains largely concentrated on broad epidemiological profiles and mortality outcomes, with limited systematic investigation into orthopedic-specific injury patterns [1, 4–6, 9, 10]. Critical data—including types of injuries, anatomical distribution, and demographic variations by age and sex—are still inadequately documented, despite their essential role in informing emergency triage protocols, surgical decision-making, and efficient allocation of medical resources.

This study aims to characterize the clinical spectrum of orthopedic injuries associated with EB that necessitate surgical management. It systematically analyzes the anatomical distribution and typological characteristics of orthopedic injuries resulting from EB, quantifies the prevalence of concomitant and multiple trauma, and investigates the associations between injury patterns and key demographic variables, including age and sex. The findings are intended to provide evidence-based insights for optimizing clinical management strategies in orthopedic practice.

## Methods

### Research design and ethical approval

This study adopted a single-center, retrospective cohort study design. The research protocol was reviewed and approved by the institutional ethics review committee (Approval No. 202602009). In accordance with relevant regulations such as the “Measures for the Ethical Review of Biomedical Research Involving Human Subjects”, and considering the non-interventional and retrospective nature of this study, the requirement for informed consent was waived.

### Data sources and patient selection

The research data were derived from the clinical database of the Hospital Information System (HIS). The inclusion period was from January 1, 2020 to December 31, 2025. A systematic screening of cases was conducted by establishing clear inclusion and exclusion criteria: the inclusion criteria were patients with a clear diagnosis who received orthopedic inpatient surgical treatment documented cause of injury includes the term “vehicle”; the exclusion criteria comprised absence of key variable data, duplicate entries, incomplete records, and injuries associated with motor vehicles or motorcycles. First, we retrieve the patient information related to vehicle-related injuries from the HIS system for all orthopedic patients. Save the results as an Excel table, including columns for PATIENT_ID, VISIT_ID, AGE, SEX, DISCHARGE_DATE_TIME, DIAGNOSIS_DESC, DEPT_NAME, and SS. The column “DIAGNOSIS_DESC LIKE” represents the cause of injury. According to the Chinese Classification and Coding of Diseases (GB/T 14396 − 2016), motorcycles and motor vehicles also have their own unique codes. Second, we conducted a meticulous review of medical records and included only those cases in which the cause of injury was explicitly documented as involving an electric bicycle.

**Fig. 1:**
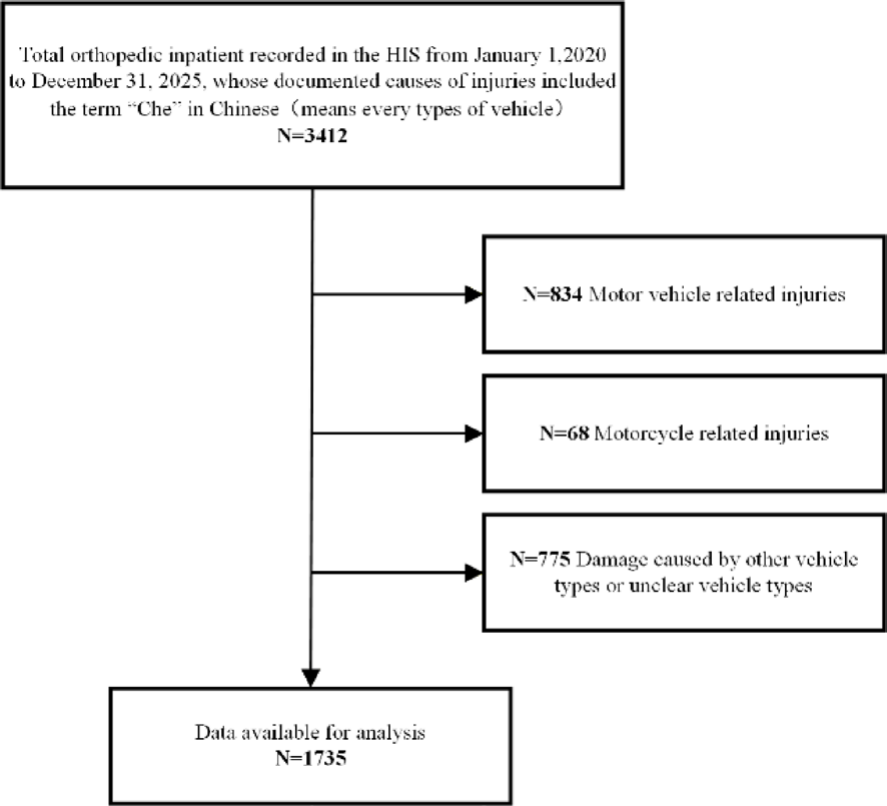
Flowchart of participants selection. HIS, Hospital information system

Thus, we acquired a dataset available for analysis, designated as “EB-related injuries,” referring to physical injuries sustained by riders, passengers, and third-party victims during the use of EB. A flowchart (Fig. 1) was used to clearly present the screening process, including the initial number of identified cases, the number of cases excluded at each stage and the reasons, and the final sample size included in the analysis, to ensure the representativeness of the study population and the transparency of data processing.

### Variable definition and classification (Core section)

Demographic variables: including age (unit: years) and gender (male/female), are used to describe the basic demographic characteristics of the research subjects.

Relevant information on accidents and injuries: Extract keywords and event descriptions related to accidents from the “DIAGNOSIS_DESC” field, summarize the types of accident mechanisms, such as frontal collision, side impact, fall, rear-end collision, etc., and standardize the classification based on this.

Injury type classification: Based on the clinical diagnostic terms in the “DIAGNOSIS_DESC” and “SS” fields, combined with orthopedic expertise, injury types are classified into six categories: fractures (F), joint dislocations (JD), meniscus or ligament injuries (M-L), nerve injuries(N), vascular injuries (V), skin and muscle injuries (S-M). The classification process involves text matching using a preset keyword dictionary (such as “fracture”, “break”, “dislocation”, “tear”, “contusion”, “hematoma”, “crush injury”, etc.), and is independently coded by two trained researchers (Cohen’s kappa = 0.92). For items with discrepancies, consensus is reached through negotiation or a third senior researcher makes the final decision to ensure the consistency and reliability of the classification results.

Anatomical site classification: Systematically categorized based on 16 predefined anatomical regions, specifically including: spine, shoulder, elbow, wrist, hip, knee, ankle, clavicle, humeral shaft, ulnar and radial shafts, pelvis, femoral shaft, tibiofibula, hand and foot, and head and brain. Each injury event is assigned to a single category based on the primary affected anatomical structure, avoiding cross-regional double counting to ensure the accuracy and operability of the classification system. Outcome variable definitions: “Combined injury (CI)” is defined as the presence of two or more different categories of injuries in the same patient at the same time.

The above outcome variables are used to quantify the complexity of individual injuries and provide a basis for subsequent analysis of injury patterns and their influencing factors.

### Statistical analyses

Descriptive statistical methods were used to summarize the basic characteristics of the study population. Continuous variables were expressed as mean ± standard deviation (SD) or median (interquartile range) depending on their distribution, fitting with Lorentzians curves and represent by center value ± width. Categorical variables were described by frequency and percentage (%). The chi-square test (χ² test) was used to compare differences in categorical variables between groups. If the expected frequency was too small, Fisher’s exact test was used. The statistical significance level was set at two-sided *p* < 0.05. All data analyses were completed using professional statistical software, including Python (version 3.9, using libraries such as pandas and scikit-learn) and SPSS (version 27.0), to ensure the reproducibility and scientific nature of the analysis process.

## Results

### Baseline characteristics of patients

We identified 1735 EB-related injuries in the orthopedic department of our hospital from January 1, 2020, to December 7, 2025, accounting for 50.85% (1735 of 3412) of all vehicle related injuries (including traffic accidents and non-traffic accidents).The following is the statistical information regarding age and gender characteristics (Table 1).

**Table 1:** Age and gender characteristics of EB-related injury patients

Gender	Age (years)							Total
	Minor	Youth		Middle	Elderly			
	0–17	18–30	31–44	45–59	60–74	75–89	≥ 90	
Male (n(%))	18 (72)	163 (67.08)	309 (71.86)	299 (51.82)	198 (50.77)	39 (56.52)	1 (100)	1027 (59.19)
Female (n(%))	7 (28)	80 (32.92)	121 (28.14)	278 (48.18)	192 (49.23)	30 (43.48)	0 (0)	708 (40.81)
Total (N(%))	25 (1.44)	243 (14.01)	430 (24.78)	577 (33.26)	390 (22.48)	69 (3.98)	1 (0.00058)	1735 (100)

Values are presented as number (%), n(%): number of individuals of a specified gender within each age group (number of individuals of a specified gender/N), N(%): number of individuals within each age group (N/number of all data). Percentages may not total 100 because of rounding

**Fig. 2:**
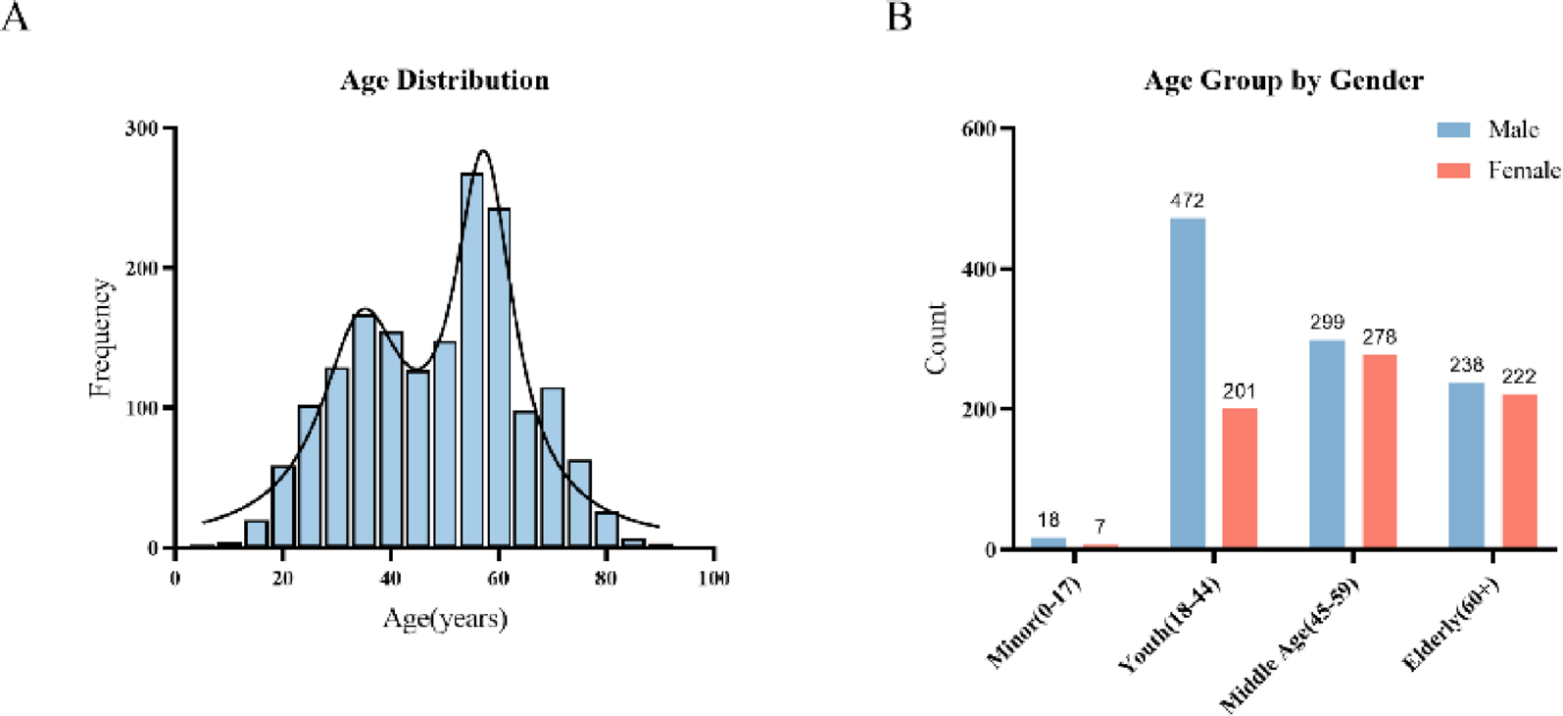
Distribution chart of age and gender. **A** Distribution histogram of age (curve: sum of two Lorentzians; Peak 1: 34.65 ± 9.41 years (95%CI: 31.70, 38.09; 95%CI: 5.95, 15.18), Peak 2 = 57.28 ± 6.72 years (95%CI: 55.98, 58.48; 95%CI: 4.20, 9.57)); **B** Cross-tabulation bar chart of age group by gender

Among the 1,735 patients, the age range was 7 to 92 years, with a mean age of 48.65 ± 15.73 years. The age distribution exhibited a bimodal pattern, with peak concentrations in two age groups: 31–44 years (*n* = 430, 24.78%) and 45–59 years (*n* = 577, 33.26%) (Fig. 2A). Notably, a substantial proportion of patients were elderly, including 390 (22.48%) aged 60–74 years, 69 (3.98%) aged 75–89 and 1 aged 92 (Table 1; Fig. 2A).

There were 1,027 male patients (59.19%) and 708 female patients (40.81%), yielding a male-to-female ratio of approximately 1.45:1 (Table 1). A cross-analysis of age and gender revealed that female patients being significantly older than male patients (female:51.55 ± 14.93 years; male: 46.64 ± 15.96 years) ( 95% CI: 3.44, 6.38; *p* < 0.001), and male population in the youth (ages 18–44) substantially outnumbers their female counterparts (Fig. 2B).

### Distribution of injury categories and anatomical sites

**Fig. 3:**
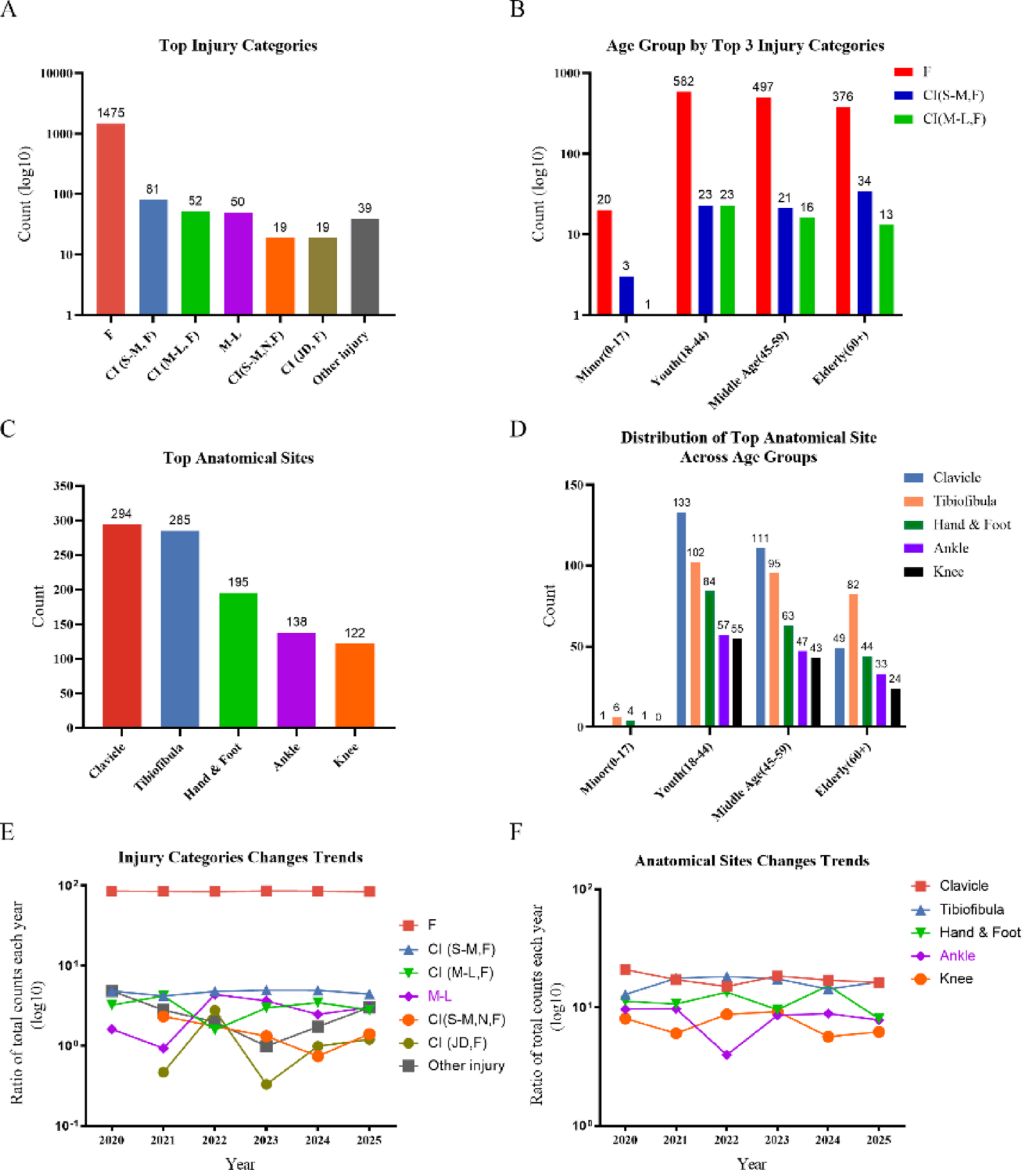
Bar charts illustrating the distribution of injury categories and anatomical sites. **A** Distribution bar chart of top 5 injury categories (including ties, F: fractures, CI: Combined injury, S-M: skin and muscle injuries, M-L: meniscus or ligament injuries, N: nerve injuries, JD: joint dislocations) ; **B** Cross-tabulation bar chart of age group by top three injury categories; **C** Distribution bar chart of anatomical site; **D** Cross-tabulation bar chart of age group by top anatomical site

This study systematically classified and analyzed all cases according to six pre-defined injury categories. As illustrated in Fig. 3A, fractures (F) constitute the predominant category of EB-related injuries, representing 85.01% (1475/1735) of all reported cases, while combined injuries rank second in frequency. Among these combined injuries, open fractures—defined as fractures accompanied by skin and muscle damage (CI(S-M, F))—are the most frequently observed (4.67%, 81/1735), followed by fractures involving meniscus or ligament injuries (CI(M-L, F)) (3.00%, 52/1735). Additional clinically significant injury patterns include open fractures (CI(S-M, F)) with concurrent nerve damage (1.10%, 19/1735) and fractures (F) occurring in conjunction with joint dislocation (JD) (1.10%, 19/1735). Further in-depth analysis of the age distribution characteristics of the three most common types of injuries reveals that among adults with simple fractures (F), the number of young people is the highest, and then decreases with age. In contrast, the number of elderly people with open fractures (CI(S-M, F)) is higher than that of other age groups (Fig. 3B).

**Fig. 4:**
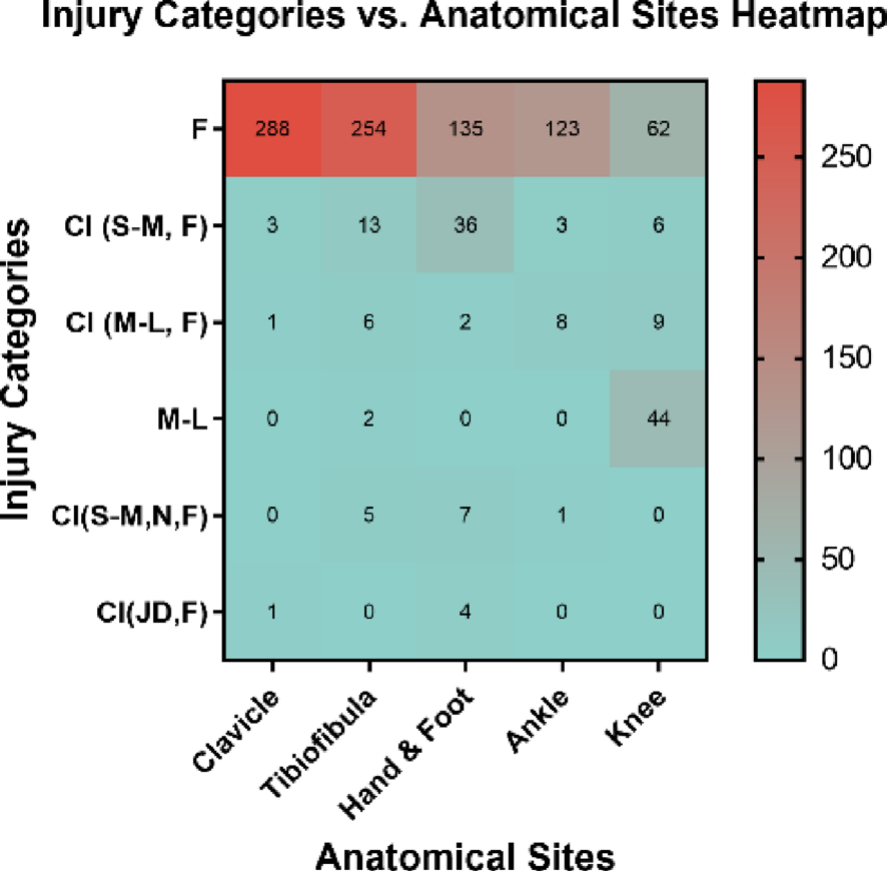
Heatmap of the top three anatomical sites cross-tabulated with top five injury categories (F: fractures, CI: Combined injury, S-M: skin and muscle injuries, M-L: meniscus or ligament injuries, N: nerve injuries, JD: joint dislocations)

In parallel, analysis of the distribution of anatomical injury sites (Fig. 3C) revealed that the most frequently affected sites were the clavicle, tibiofibula, hand and foot, ankle joint and knee joint. Further evaluation demonstrated that clavicle fractures were the most prevalent in the young adult cohort (19.76%, 133/673), whereas tibiofibular fractures exhibited a consistently high incidence across all age groups (Fig. 3D). Except for meniscus or ligament injuries (M-L), which has increased since 2022 compared to before, the annual change trends of other top injury types have been relatively stable. Fractures (F) and open fractures (CI(S-M, F)) are always in the top1 and top2 positions (Fig. 3E). Correspondingly, the annual change trend of the Top anatomical sites is also relatively stable (Fig. 3F). The cross-analysis of injury types and anatomical sites of injury (Fig. 4) showed that among combined injuries, open fractures (CI(S-M, F)) are most common in the hand and foot (18.46%, 36/195), while meniscus or ligament injuries (M-L) are most common in the knee (36.07%, 44/122). Besides meniscus or ligament injuries (M-L), open fractures (CI(S-M, F)) and fractures involving meniscus or ligament injuries (CI(M-L, F)) are also common in the knee.

## Discussion

This single-center retrospective study systematically characterizes the orthopedic profile of EB-related injuries that necessitate surgical management over a nearly six-year period. Analysis of 1,735 cases reveals distinct epidemiological patterns, injury characteristics, and demographic variations with substantial implications for clinical practice.

The subsequent discussion scrutinizes these findings and endeavors to convert them into actionable insights to direct clinical decision - making and optimize resource allocation for orthopedic surgeons dealing with patients having EB - related injuries who have completed the initial triage and are in need of surgical treatment. It is crucial to emphasize that the follow clinical hypotheses formulated in this study are grounded in retrospective data. Future multicenter prospective studies are required to obtain robust evidence that can guide clinical practice.

### The shifting demographics of trauma: highlighting the vulnerability of middle-aged and elderly populations

A pivotal finding of this study is the bimodal age distribution of EB-related injuries, with peaks in the late youth age (31–44), and more prominently, the middle age groups (45–59), along with a substantial proportion (> 23%) of patients aged 60 years or older. This demographic pattern stands in marked contrast to the conventional understanding of road traffic injuries, which have traditionally predominated among younger adults [11–13]. The observed shift is primarily driven by the widespread adoption of EB as a primary mode of daily transportation among middle-aged and elderly individuals for commuting, shopping, and other routine activities [14, 15].

This activity was not affected by the COVID-19 pandemic. Our data shows that during the global lockdowns from 2020 to 2022, only in 2020, when the pandemic was at its peak and lockdowns were the strictest, did the data show a significant decrease. The overall number of cases during the three years of the pandemic remained consistent with the trend in the three years after the pandemic (Supplementary Table 1). Based on the knowledge we have, we believe that this is because e-bikes, being “contactless and short-distance”, became the preferred alternative to public transportation. At the same time, we recognize that due to this study being a single-center study, the impact of the pandemic on mobility patterns and injury rates needs to be verified by further research.

*Clinical hypotheses 1*: Based on our findings, these results may suggest the potential utility of refining initial assessment and triage protocols for EB-related trauma patients. Specifically, clinical practice could consider incorporating a heightened vigilance for polytrauma in middle-aged and older adults—particularly when the reported injury mechanism seems minor—as our data indicate a higher prevalence of complex injuries in this subgroup. Additionally, emergency department triage systems might benefit from recognizing age ≥ 45 years as a factor that warrants more comprehensive evaluation, potentially including a lower threshold for advanced imaging (e.g., CT or MRI) to detect occult fractures or associated injuries. However, these proposed adjustments require prospective validation in diverse clinical settings before widespread implementation.

### The high proportion of fractures and combined injuries: potential indicators of substantial energy transfer

Our data unequivocally demonstrate that fractures account for the vast majority (85.01%) of EB-related orthopedic injuries. More significantly, the high prevalence of combined injuries (CIs), particularly open fractures (4.67%) and fractures accompanied by ligamentous damage (3.00%) - may suggest substantial energy transfer during EB accidents (Fig. 3A, B), relative to conventional bicycles. While we lack direct biomechanical measurements (e.g., collision speed, impact force), this injury pattern aligns with prior reports of EB-related trauma complexity [1, 16]. Due to the lack of detailed collision mechanisms and speed data, the strength of our inference on the damage mechanism is limited, and it also affects the ability to conduct reliable multivariate regression analysis to determine independent risk factors. Therefore, in future prospective studies, detailed information such as collision mechanisms, speed, and the use of protective equipment should be systematically collected to conduct multivariate analysis and determine the independent predictors of injury severity, thereby better guiding clinical practice.

*Clinical hypotheses 2*: Given the notably high incidence of complex injuries observed in our cohort, a structured, multidisciplinary response upon admission may be worth considering. Building on our findings, we explore the potential utility of a standardized “EB Trauma Workup Process” for admitted patients. The final effect will be systematically evaluated as part of future prospective studies, which may include the following elements.

*Primary and secondary physical examination*: Rigorous adherence to Advanced Trauma Life Support (ATLS) [17] guidelines would likely ensure prompt identification of life‑threatening conditions and systematic assessment to reduce the risk of missed injuries—a practice already standard in trauma care but potentially under-emphasized for EB-specific contexts.

*Systematic imaging protocol*: A mechanism-guided, whole-body radiographic evaluation could prioritize anatomical regions most frequently affected in our study (clavicle, tibiofibula, hand/foot, ankle, knee). Proactive use of computed tomography (CT) for these areas of clinical concern may improve precision in defining fracture architecture and detecting concomitant dislocations or soft tissue injuries, though its cost-benefit ratio requires further evaluation.

*Early specialist consultation*: Early engagement of microsurgical orthopedics for open fractures (CI(S-M, F)) and of sports medicine for suspected ligamentous injuries (CI(M-L, F)) could plausibly facilitate timely surgical debridement and reconstruction. This approach may help minimize complications and optimize long-term functional outcomes, but prospective studies are needed to confirm its effectiveness in diverse clinical settings.

### Anatomical site specificity: informing diagnostic focus and surgical preparedness

The analysis of anatomical injury distribution reveals a well-defined pattern of vulnerability, reflecting distinct biomechanical mechanisms inherent to EB accidents [18]. The high incidence of clavicle, tibiofibular, and hand/foot injuries underscores characteristic force transmission pathways during collision events (Figs. 3B and C and 4). Clavicle fractures—most prevalent among younger adults—are strongly associated with direct impact from handlebars or falls onto the shoulder girdle. Tibiofibular fractures, consistently observed across all age groups, correspond to the “bumper” effect and direct axial loading of the lower extremity. The frequent involvement of the hands and feet suggests protective reflexive movements during falls or crush mechanisms due to the vehicle’s weight upon impact.

*Clinical Hypotheses 3*: This site-specific injury profile may support a more strategic and efficient clinical evaluation for EB-related injuries. Clinicians could consider implementing a standardized “E-bike Injury Focused Examination” protocol—tailored to the high-risk anatomical regions identified in our study—to complement routine trauma assessments, even in the presence of concomitant major trauma. The proposed operational details of the “E-bike Injury Focused Examination” may include follows elements.

*Systematic palpation sequence*: Prioritize high-frequency injury sites in a fixed order: (1) Clavicle: Palpate the midshaft and lateral third (common fracture zones) for tenderness/deformity; (2) Knee: Assess medial/lateral joint lines (meniscus) and tibial plateau (fracture); (3) Ankle/Wrist: Palpate malleoli (ankle) and distal radius (wrist) for bony tenderness; (4) Foot/Hand: Check metatarsals (foot) and metacarpals (hand) for localized pain/swelling.Each region should be palpated bilaterally for comparison.

*Targeted stress testing (e.g.*,* Knee injuries)*: Given the knee’s susceptibility to meniscal/ligamentous injuries in our cohort, a focused ligamentous assessment may be warranted if clinical suspicion exists: 1)Lachman Test: Evaluate anterior cruciate ligament (ACL) integrity (knee flexed 30°, anterior drawer motion >3 mm = positive); 2) Anterior Drawer Test: Confirm ACL stability (knee flexed 90°, tibia pulled forward); 3) Varus/Valgus Stress Tests: Assess medial/lateral collateral ligament stability (valgus stress at 0°/30°flexion). Positive tests should trigger targeted imaging (MRI/CT) rather than routine use in all patients.

Preoperative Planning Considerations: For common fractures (e.g., clavicle, tibiofibula), standardizing preoperative workflows might enhance surgical readiness. This could include: (1) Pre-stocking OR trays with implants sized for our study population (e.g., 3.5 mm locking plates for clavicle, 8–10 mm intramedullary nails for tibiofibula); (2) Routine review of imaging for fracture comminution (to anticipate need for bone graft or external fixation). These measures may reduce intraoperative delays and improve care coordination, but their cost-effectiveness requires validation.

*Note*: All recommendations should be adapted to individual patient factors (e.g., age, comorbidities) and require prospective validation in diverse clinical settings before widespread adoption.

### Gender and age interactions: tailoring patient communication and rehabilitation

The finding that female patients were significantly older, while young males constituted a disproportionately large proportion of trauma cases (Table 1; Fig. 2B), highlights the necessity of age- and sex-specific approaches to patient management. The proportion of young male individuals was higher in this study (472 cases of males aged 18–44, accounting for 70.13% of the total cases in this age group). In combination with previous literature reports [11–13](the document does not elaborate on this point, but based on my knowledge, young men are more prone to risky cycling behaviors such as speeding and running red lights), it is speculated that their high-risk behaviors may increase their susceptibility to trauma. However, this study did not directly measure cycling behaviors, and further research is needed for verification. In contrast, older female patients, who are at higher risk of osteopenia or osteoporosis, are more vulnerable to fractures even from low-energy mechanisms such as simple falls [19, 20].

*Clinical Hypotheses 4*: Clinical management could potentially benefit from integrating personalized strategies in both patient education and rehabilitation planning, tailored to the distinct injury patterns observed in our cohort. For young male patients—who accounted for a notable proportion of EB-related injuries cases in our study—structured counseling on injury prevention and safe riding practices during discharge may be a valuable addition to standard care. For older female patients, care might extend beyond acute injury management to address underlying risk factors. Our data showing a higher prevalence of fragility-related fractures in this subgroup suggests that targeted fall prevention measures—coupled with systematic assessment for osteoporosis—could be considered. Incorporating dual-energy X-ray absorptiometry (DEXA) scanning and endocrinology referral may help mitigate long-term recurrent fracture risk, though the cost-effectiveness of this approach requires validation in prospective studies.

When contextualizing our findings within the global landscape of EB-related injuries, several notable contrasts and parallels emerge. A study from the Netherlands, a nation with extensive cycling infrastructure, found that EB accidents accounted for 26.7% of all fatal bicycle accidents, and EB-related injuries patients were older, had higher mortality rates, and were more likely to sustain polytrauma compared to classic bicycle users [21]. This aligns with our observation of a high proportion of middle-aged and elderly patients, though our study’s focus on surgically managed orthopedic injuries precludes direct mortality comparisons. In North America, the epidemiological trend points toward a sharp increase in EB-related injuries, particularly among younger populations. A U.S. national study reported a 293% increase in the population-based rate of e-bike injuries from 2019 to 2022 [22]. Furthermore, a pediatric-focused study presented at the American Academy of Orthopaedic Surgeons indicated a surge in e-bike injuries requiring trauma activation among youth from 2017 to 2023, with these patients sustaining more extremity injuries and fractures compared to those in pedal bike accidents [23]. This contrasts with our cohort’s bimodal age distribution peaking in middle age, potentially reflecting differences in riding demographics, regulations, or exposure patterns between China and the U.S. Across other Asian regions, EB-related injuries constitute a major public health burden. The World Health Organization reports that bicyclists (including e-bike riders) account for approximately 12% of all road traffic fatalities in the South-East Asia Region [24]. A systematic review of the Chinese context noted that the mortality rate of bicycle and e-bike-related injuries increased from 4.86% in 2000 to 6.97% in 2019 [25].

These international comparisons highlight that while the rising injury burden associated with EB is a global phenomenon, the demographic profiles, injury mechanisms, and policy responses exhibit regional specificity. Our data from Anhui Province, China—showing a high prevalence of fractures (85.01%) and a vulnerable middle-aged/elderly population—contributes a distinct epidemiological profile to this global discussion. The consistency in observing severe injury patterns (e.g., polytrauma, high fracture rates) across diverse settings reinforces the need for targeted clinical protocols and preventive strategies tailored to local riding populations and infrastructure realities.

Pursuant to China’s Hospital Classification System and the Hierarchical Diagnosis and Treatment Policy, our institution is a Grade III, Class A hospital—designated to provide comprehensive care to the entire local population, including both military and civilian residents. Following triage and initial assessment in the emergency department, orthopedic specialists assume responsibility for subsequent clinical evaluation, including mechanism-based history taking, focused physical examination, interpretation of imaging studies, and surgical management as indicated. For case identification, we employ a dual-method approach integrating electronic health record data extraction from the Hospital Information System (HIS) with systematic manual review of medical charts, thereby optimizing case ascertainment accuracy and minimizing both false-positive and false-negative classification errors. ( False-positive: Errors in the doctor’s medical records may lead to injuries from pedal bicycles or other non-motorized vehicles being mistakenly marked as related to electric bicycles. False-negative: The injury factors were not detailedly recorded in the medical records, resulting in the electric vehicle-related injuries being wrongly marked as “vehicle type unknown”.)However, due to the requirements of the national medical insurance department and the hospital medical record department for the quality of medical records, each of our medical records is regularly inspected and scored by a third party. This system ensures that the proportion of classification errors will be very low.

*Other limitations of this study include*: (1) Inability to differentiate injury roles in e-bike collisions—specifically riders, passengers, or third-party victims—due to the absence of role-specific data in the ‘HISDIAGNOSIS_DESC’ field, potentially confounding injury mechanism interpretation (e.g., impact trauma in third-party victims versus loss-of-control injuries in riders); (2) Absence of prospectively collected functional outcomes (e.g., postoperative range of motion), time-to-recovery metrics (e.g., return to work or sport), and long-term sequelae (e.g., post-traumatic arthritis), precluding robust evaluation of clinical effectiveness; (3) Potential selection bias arising from the inclusion of non-surgical patients referred to our center due to deviations from the hierarchical care protocol; (4) Lack of standardized injury severity quantification (e.g., using the Injury Severity Score [ISS] or AO/OTA classification system), limiting cross-study comparability of injury burden; and (5) Residual inter-rater variability in narrative text coding, despite implementation of a dual-independent coding protocol with third-party arbitration.

## Conclusions

In summary, this study maps a clinical surgical spectrum of EB-related injuries that is characterized by an older patient demographic, a high preponderance of fractures and complex combined injuries, and specific anatomical vulnerabilities. By integrating these evidence-based findings into clinical practice—through refined triage protocols, systematic diagnostic bundles, focused examinations, and personalized patient management—clinicians can improve the quality and efficiency of care for this growing patient population. Future efforts should focus on developing and validating these proposed clinical pathways to ultimately enhance patient outcomes. This study has several limitations, including its retrospective, single-center design, which may limit the generalizability of the findings. The reliance on clinical database coding also introduces the potential for misclassification bias, despite our rigorous text-matching and dual-coding strategy. Prospective, multi-center studies are warranted to further validate our conclusions.

## Electronic supplementary material

Below is the link to the electronic supplementary material.

Supplementary file 1 (XLSX 55 kb)

## Data Availability

The data generated in this study are available from the corresponding author upon reasonable request.
